# Unlocking New Reactivities in Enzymes by Iminium Catalysis

**DOI:** 10.1002/anie.202203613

**Published:** 2022-06-15

**Authors:** Guangcai Xu, Gerrit J. Poelarends

**Affiliations:** ^1^ Department of Chemical and Pharmaceutical Biology Groningen Research Institute of Pharmacy University of Groningen Antonius Deusinglaan 1 9713 AV Groningen The Netherlands

**Keywords:** Biocatalysis, Chemomimetic Catalysis, Iminium Ions, Organocatalysis

## Abstract

The application of biocatalysis in conquering challenging synthesis requires the constant input of new enzymes. Developing novel biocatalysts by absorbing catalysis modes from synthetic chemistry has yielded fruitful new‐to‐nature enzymes. Organocatalysis was originally bio‐inspired and has become the third pillar of asymmetric catalysis. Transferring organocatalytic reactions back to enzyme platforms is a promising approach for biocatalyst creation. Herein, we summarize recent developments in the design of novel biocatalysts that adopt iminium catalysis, a fundamental branch in organocatalysis. By repurposing existing enzymes or constructing artificial enzymes, various biocatalysts for iminium catalysis have been created and optimized via protein engineering to promote valuable abiological transformations. Recent advances in iminium biocatalysis illustrate the power of combining chemomimetic biocatalyst design and directed evolution to generate useful new‐to‐nature enzymes.

## Graphical abstract

This Minireview summarizes the different strategies used in the design and engineering of novel enzymes that accommodate iminium catalysis. The advantages and challenges of developing enzymes for this catalysis mode are discussed. Recent developments in iminium biocatalysis showcase the tremendous power of combining chemomimetic biocatalyst design and directed evolution to create useful biocatalysts for new‐to‐nature transformations.

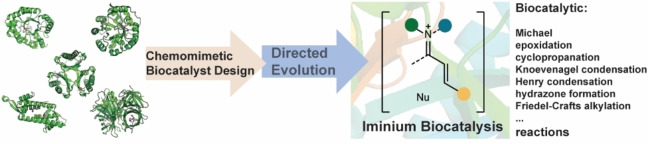

## 1 Introduction

The use of nature's catalysts, enzymes, for chemical synthesis has evolved from an academic curiosity to a key applied technology.[^1^, ^2^, ^3^] Developing new enzymes is a central topic in biocatalysis, essential for enriching the biocatalytic toolbox used for elegant biocatalytic retrosynthesis design.[^4^, ^5^, ^6^] While natural enzyme discovery (e.g. (meta)genome mining) continues to be an important source for new biocatalysts, the enzymes nature offers are often limited to catalyzing physiologically relevant reactions. So far, the types of reactions used in applied biocatalysis are dwarfed by the vast number of reactions catalyzed by small‐molecule catalysts (i.e., transition‐metal catalysts and organocatalysts). Especially for reactions like the construction of C−C bonds, which is fundamental to organic synthesis, widely applicable enzymes are rare.[^7^, ^8^] Hence, developing enzymes that promote synthetically useful non‐natural reactions is highly important. Towards this end, enzyme engineers have taken inspiration from the advancements in small‐molecule catalysis.

Interestingly, many of the small‐molecule catalysts were initially bio‐inspired;^[9]^ for instance, the catalytic mechanism of class I aldolases inspired the use of proline in amine catalysis.^[10]^ Further design and optimization of small‐molecule catalysts by synthetic chemists greatly expanded their reaction scope and preparative usefulness. Transferring such catalytic systems back to enzyme platforms has become an emerging strategy to create new‐to‐nature enzymes with great synthetic utility.^[11]^ The potential of such a chemomimetic strategy is nicely illustrated by several elegant studies reported by Arnold and co‐workers, who have engineered a panel of novel heme‐dependent enzymes that mimic metal‐catalyzed carbene and nitrene transfer reactions,^[12]^ promoting stereoselective C−C,[^13^, ^14^] C−N,^[15]^ C−Si,^[16]^ and C−B^[17]^ bond‐forming reactions.

**Figure 1:**
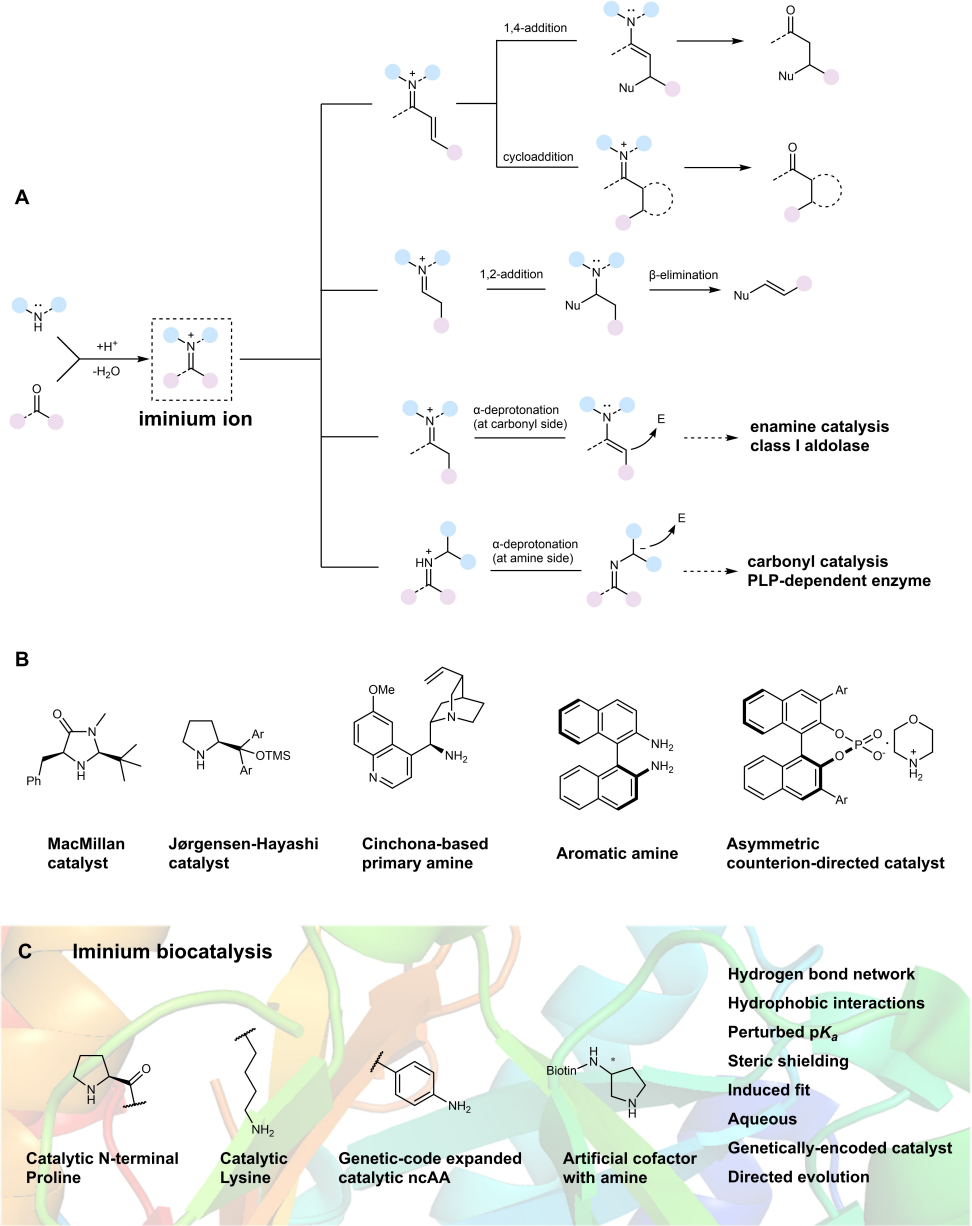
A) Typical reaction pathways of iminium catalysis, enamine catalysis, and carbonyl catalysis. B) Example of common organocatalysts used for iminium catalysis. C) Current approaches for the development of enzymes for iminium biocatalysis.

Organocatalysis has become a powerful method for asymmetric synthesis (highlighted by the 2021 Nobel Prize in Chemistry^[18]^), forming the third pillar of asymmetric catalysis in addition to transition‐metal catalysis and biocatalysis.[^19^, ^20^] The founding branch of organocatalysis, amine catalysis, has a myriad of transformations but was initially built on only two essential catalysis modes: enamine and iminium catalysis.[^21^, ^22^, ^23^] Whereas enamine catalysis is employed by nature (e.g. class I aldolases), iminium catalysis by natural enzymes is uncommon. Notably, an iminium ion may be used in reactions catalyzed by enzymes such as imine reductase and Pictet–Spenglerases,[^24^, ^25^] but here the iminium ion is not the result of substrate activation by an amine catalyst. Recently, several captivating enzyme systems have been developed to promote reactions where iminium ions are formed as catalytic intermediates. In this Minireview, we summarize recent developments in iminium biocatalysis (last five years), with a focus on the diverse strategies employed in engineering new enzymes for iminium catalysis. For organocatalytic iminium catalysis, we refer the readers to several outstanding reviews.[^22^, ^26^, ^27^] Although iminium ion formation constitutes part of the catalytic cycle of PLP‐dependent enzymes (e.g. threonine aldolases) and class I aldolases, we consider these reaction types to be carbonyl catalysis[^28^, ^29^] and enamine catalysis, respectively (Figure 1A); hence, they are excluded from this review. We first give a brief overview of iminium catalysis and discuss the advantages and challenges of developing enzymes for this catalysis mode. Subsequently, we discuss in more detail the different strategies used in constructing and engineering various novel enzymes that accommodate iminium catalysis.

## 2 Iminium Biocatalysis

Although sporadic cases have been reported for over one hundred years, it was only in 2000 that MacMillan and co‐workers coined the term “organocatalysis” and introduced the modern concept of iminium catalysis.[^30^, ^31^, ^32^] Along with enamine catalysis realized by List, Lerner, and Barbas III at the same time,^[10]^ iminium and enamine catalysis ignited a highly intensive “gold rush” period in organocatalysis research during the first decade of the 21^st^ century. Iminium catalysis can be promoted using both secondary and primary amines (Figure 1B), with secondary amines tending to dominate the field. The catalysis is achieved through the reversible formation of an iminium ion intermediate between the carbonyl substrate and amine catalyst (Figure 1A). This intermediate is more electrophilic than the corresponding aldehyde or ketone due to the LUMO‐lowering effect similar to Lewis acid catalysis. Depending on the nucleophiles and catalysts used, the iminium ion intermediate can undergo various reaction pathways such as 1,4‐addition, cycloaddition, and 1,2‐addition, giving rise to a broad range of synthetically useful chemistries (Figure 1A).^[22]^ A plethora of popular catalysts have been developed, their stereoselectivities are usually accomplished by steric hindrance and/or hydrogen bonding interactions (Figure 1B).[^27^, ^33^, ^34^, ^35^, ^36^, ^37^]

Developing new‐to‐nature enzymes that adopt the iminium catalysis mode can offer several unique advantages compared to small‐molecule catalysts (Figure 1C). To begin with, enzymes offer exquisite reaction pathway control. Orchestrated by the teamwork of the catalytic residues, the enzyme active site can provide multiple interactions with substrates simultaneously via hydrogen‐bond networks, hydrophobic interactions, and steric shielding, usually resulting in excellent product selectivities. Second, enzymes are dynamic proteins that undergo conformational changes to facilitate the various steps during iminium catalysis, e.g. substrate recruitment, iminium ion formation, and product release, which can potentially confer higher catalytic efficiency, allowing low catalyst loading. Biocatalysis also uses water as a benign solvent, greatly reducing the usage of toxic organic solvents, and enzymes can often be easily produced by bacterial fermentation and are biodegradable. Finally, directed evolution has emerged as a powerful method for biocatalyst optimization.[^38^, ^39^, ^40^]

Despite the promising aspects, designing iminium biocatalysts is a challenging task. Enzymes that naturally perform iminium catalysis are uncommon and only a few enzymes possess a catalytic amine. The covalent catalysis nature of iminium catalysis results in multiple covalent reaction intermediates, further increasing the difficulty of the design. Although directed evolution is a valuable and relatively straightforward method of biocatalyst optimization, the identification of a starting activity for evolution is less predictable. Recent progress has offered some exciting approaches to develop iminium biocatalysts. Based on catalytic promiscuity, researchers have repurposed both natural and computationally designed enzymes containing a catalytic N‐terminal proline or lysine for iminium biocatalysis. In addition, artificial enzymes with a catalytic aniline or a synthesized amine‐containing cofactor have been designed to accommodate iminium catalysis (Figure 1C).

## 3 Repurposing Natural Enzymes

Catalytic promiscuity is an important source for new biocatalysts.[^7^, ^41^, ^42^, ^43^] Given the success of secondary and primary amines as organocatalysts in iminium catalysis, Poelarends and co‐workers have explored and optimized two natural enzymes having such key catalytic amines for iminium biocatalysis: 4‐oxalocrotonate tautomerase (4‐OT) and 2‐deoxyribose‐5‐phosphate aldolase (DERA).

### 3.1 4‐Oxalocrotonate Tautomerase (4‐OT)

**Figure 2:**
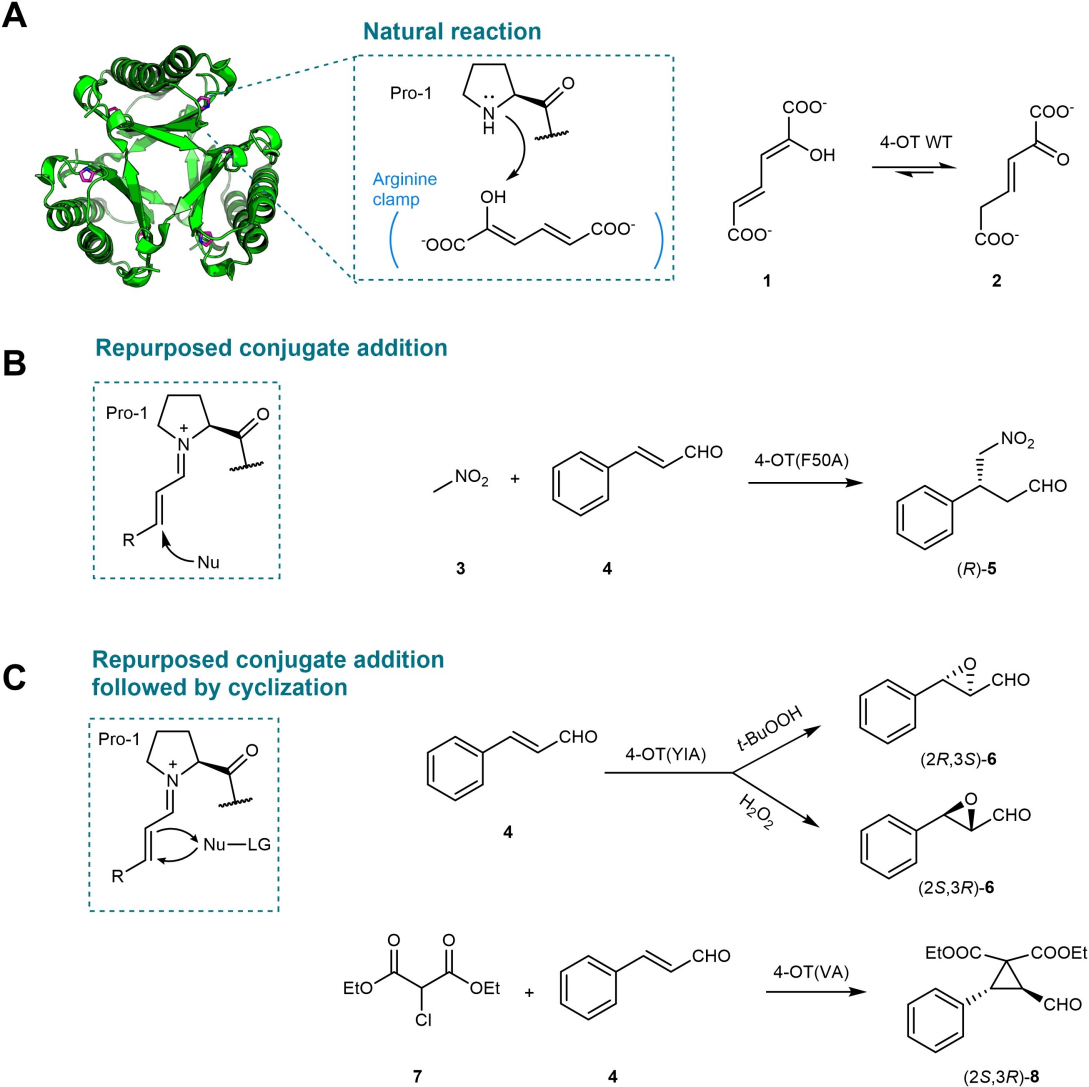
Repurposing 4‐OT for iminium biocatalysis. A) The native reaction of 4‐OT. B) Michael addition catalyzed by 4‐OT(F50A). C) Cycloaddition‐type reactions (epoxidation and cyclopropanation) catalyzed by 4‐OT mutant enzymes.

The enzyme 4‐OT is part of a bacterial pathway for the degradation of aromatic hydrocarbons.^[44]^ It belongs to the tautomerase superfamily (TSF), a group of enzymes that share a characteristic β‐α‐β building block and often possess an unusual catalytic N‐terminal proline (Pro‐1).^[45]^ Inspired by the use of proline and other secondary amine catalysts in organocatalysis, Poelarends and co‐workers tested whether these 4‐OT enzymes can be used for amine catalysis. Indeed, the homohexameric 4‐OT from *Pseudomonas putida* mt‐2 with a monomer size of only 62 amino acids was found to be a good candidate. It naturally catalyzes the tautomerization of 2‐hydroxymuconate (**1**) to yield 2‐oxohex‐3‐enedioate (**2**, Figure 2A). The key catalytic residue Pro‐1 functions as a base to abstract the proton from the hydroxyl group, while several arginine residues (Arg‐11, Arg‐39, and Arg‐61) form an arginine clamp for substrate binding.^[45]^ Previously, this catalytic N‐terminal proline was redirected to act as a nucleophile and promote enamine catalysis. Using 4‐OT as a template, biocatalytic Michael‐type addition reactions of simple aldehydes to nitroolefins and aldol reactions between two aldehydes were developed, giving access to valuable pharmaceutical precursors such as γ‐nitroaldehydes and 1,3‐diols.[^46^, ^47^, ^48^, ^49^, ^50^, ^51^] By taking advantage of the small monomer size of 4‐OT, a collection of 4‐OT genes encoding nearly all possible single‐mutant variants was constructed for systematic mutagenesis studies and rapid identification of “hotspot” positions for protein engineering.^[52]^

More recently, the catalytic site of 4‐OT was engineered to unlock iminium catalysis, giving rise to new promiscuous activities. Poelarends and colleagues envisioned and found that 4‐OT can catalyze the enantioselective Michael addition of nitromethane (**3**) to various α,β‐unsaturated aldehydes via enzyme‐bound iminium ion intermediates, providing a new biocatalytic route to the desired enantiomer of pharmaceutically relevant γ‐nitroaldehydes (Figure 2B).^[53]^ Screening of the 4‐OT single‐mutant collection^[52]^ led to 4‐OT(F50A) with about 10‐fold specific activity enhancement for the Michael addition of nitromethane (**3**) to cinnamaldehyde (**4**). Various α,β‐unsaturated aldehydes were accepted by 4‐OT(F50A), furnishing the corresponding γ‐nitroaldehydes in good yield and with high enantiopurity (up to 98 % *ee*).^[53]^ The formation of enzyme‐bound iminium ion intermediates between the catalytic Pro‐1 and the α,β‐unsaturated aldehydes was supported by labeling and mass spectrometry experiments and the loss of function in the proline knock‐out construct (P1A). Compared to the well‐established Jørgensen–Hayashi catalyst,^[54]^ 4‐OT(F50A) has about 7 times higher catalytic turnover and enables reactions in aqueous buffer instead of organic solvent (Table 1).

**Table 1:** An overview of different catalytic systems for the Michael addition of nitromethane to α,β‐unsaturated carbonyls.^[a]^

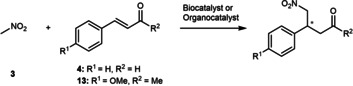							
							
	Substrate loading	Nucleophile [equiv]	Solvent	Catalyst loading [mol %]	TTN^[b]^	Isolated yield [%]	Product *ee* [%]
Jørgensen–Hayashi catalyst	500 mM **4**	3.0	MeOH	10	10 (16 h)	90	95
4‐OT (F50A)	25 mM **4**	2.0	HEPES/EtOH (95/5)	1.4	70 (11 h)	85	96
4‐OT (F11)	40 mM **4**	1.5	HEPES/EtOH (95/5)	0.08	1250 (24 h)	65	98
DERA‐MA	5 mM **4**	4.0	HEPES/DMSO (97/3)	0.08	1250 (16 h)	66	98
RA95.5‐8 (T53L/K210H)	2 mM **13**	25.0	HEPES/MeCN (98.8/1.2)	0.05	2000 (24 h)	82	92
**Sav‐32**	6.6 mM **4**	5.0	KP_i_/MeOH (50/50)	1.0	80 (18 h)	80^[c]^	82

[a] Examples selected based on Ref. [53, 54, 58, 64, 75, 98]. Reaction conditions displayed are based on the preparative‐scale experiments. [b] TTN (total turnover number) was calculated based on the catalyst loading and the conversion of the reaction; time in brackets is the total reaction time. [c] Crude yield by NMR analysis.

The synthetic versatility of iminium catalysis also inspired the same researchers to build new activities into 4‐OT. In addition to Michael additions, when nucleophiles attached to a leaving group are applied, conjugation addition followed by intramolecular cyclization can be realized (Figure 2C). Xu et al. reported the engineering of 4‐OT into an unusual cofactor‐independent peroxygenase which can promote enantiocomplementary epoxidation reactions using either *t‐*BuOOH or H_2_O_2_ as the nucleophile.^[55]^ The enzyme‐bound iminium ion can be initially attacked by the hydroperoxide, resulting in an enamine intermediate which can undergo ring closure to release either *t‐*BuOH or water to form the epoxide moiety (Figure 2C). Screening of the collection of 4‐OT single mutants rapidly identified several “hotspot” positions, which were targeted by iterative site‐saturation mutagenesis. A triple‐substituted variant 4‐OT(YIA, Q4Y/M45I/F50A) was obtained, displaying a 60‐fold improved specific activity compared to wild‐type 4‐OT towards the epoxidation of cinnamaldehydes using *t‐*BuOOH as the oxidant. Interestingly, when switching the oxidant to H_2_O_2_ using the same enzyme, the enantio‐preference of the epoxides formed was inverted compared to *t‐*BuOOH. Notably, this switching of enantioselectivity was not observed in organocatalysis. Later, the catalytic repertoire was further expanded to include cyclopropanation reactions in a similar paradigm. Kunzendorf et al. showed that diethyl 2‐chloromalonate (**7**) can also be used as a nucleophile to attack the enzyme‐bound iminium ion, followed by dechlorination to form a cyclopropane ring (Figure 2C).^[56]^ In this case, the success of the cyclopropanation reaction also depends on the tuning of the leaving group ability. Replacing the chlorine by a weak leaving group (fluorine), results in a Michael addition reaction whereas the use of a stronger leaving group (bromine) led to the inactivation of the enzyme by the alkylation of the catalytic proline. Using the same mutability landscape data‐driven approach,^[52]^ a double‐substituted mutant 4‐OT(M45V/F50A) with 30‐fold enhancement in specific activity was engineered. A set of cyclopropanes was prepared with good to excellent enantioselectivity (*ee* up to 98 %) and diastereoselectivity (dr up to 25 : 1).^[56]^

One of the promising aspects of developing iminium biocatalysts is the potential unparalleled catalytic efficiency of enzymes. While the catalytic machinery of 4‐OT is malleable for iminium catalysis and limited protein engineering efforts permitted lower catalyst loading (around 0.5–1 mol %) compared to organocatalysts (typically 10–20 %), the catalytic efficiencies reported are still dwarfed by natural enzyme activities (Table 1). Considering that natural evolution took countless years to create exquisite enzyme active sites, a more extensive laboratory evolution campaign is needed to boost these promiscuous iminium catalysis activities. However, the symmetry relationship within homohexameric 4‐OT, with any point mutation reflected in all six subunits, imposes a significant limitation for its genetic optimization. Inspired by the gene duplication and fusion strategy nature used to make new enzymes within the TSF,[^45^, ^57^] Xu et al. designed and evolved a tandem fused 4‐OT for the enantioselective Michael addition of nitromethane to cinnamaldehyde.^[58]^ The head‐to‐tail topology of the 4‐OT dimer allowed the connection of the C‐ and N‐terminus of two 4‐OT monomers via a short fusion linker, GGGAG. This tandem fused 4‐OT has a reduced symmetry compared to “unfused” wild‐type 4‐OT, expanding its genetic optimization potential by allowing independent sequence diversification of neighboring subunits, and thus enlarging the protein sequence space that can be explored by directed evolution. After eleven rounds of directed evolution, the evolved variant 4‐OT(F11) displayed a 320‐fold enhancement in catalytic activity, promoting the Michael addition of nitromethane to cinnamaldehyde with a respectable catalytic efficiency *k*_cat_/*K*_M_ of 2.8×10^3^ M^−1^ s^−1^, enabling the gram‐scale synthesis of the desired (*R*)‐γ‐nitroaldehyde product **5**. Compared to the previously constructed single‐mutant F50A, 4‐OT(F11) enables the usage of a higher concentration of **4**, using only 1.5 equivalent of **3** as well as a considerably lower catalyst loading (0.08 mol %, Table 1). The applied fusion strategy may also find application in the efficient engineering of other oligomeric enzymes.

### 3.2 Deoxyribose‐5‐phosphate Aldolase (DERA)

**Figure 3:**
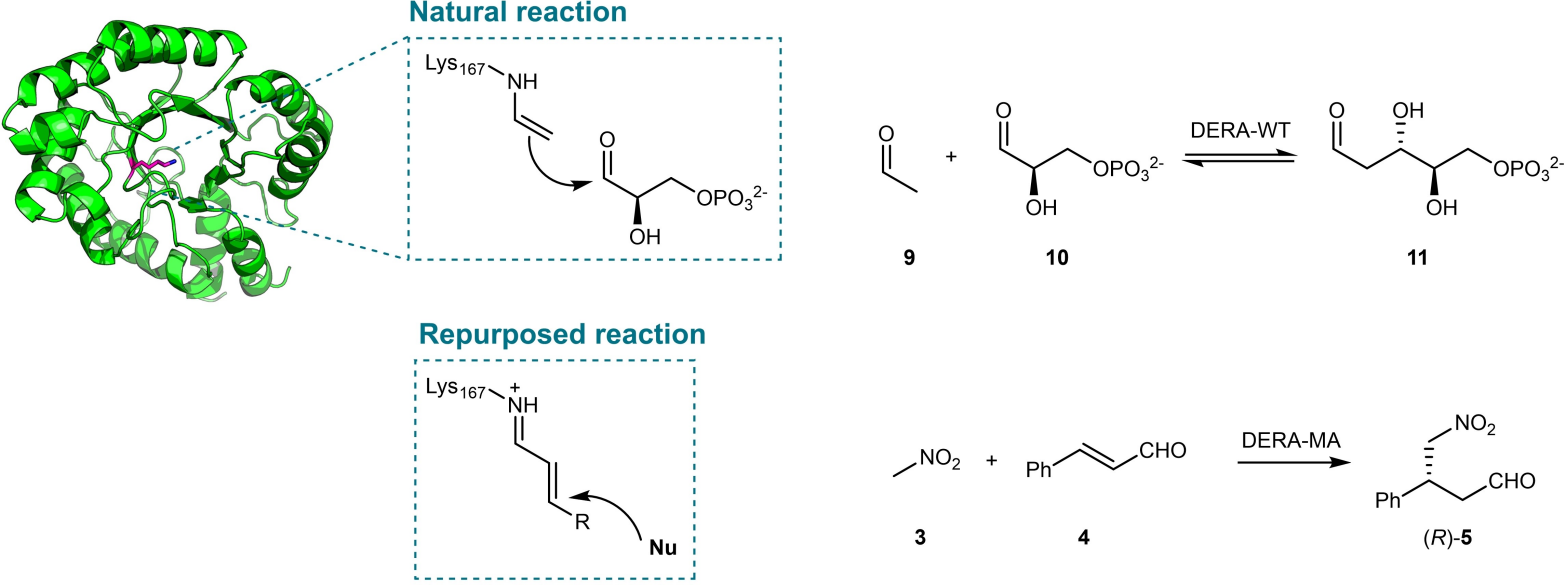
The natural aldol reaction and repurposed Michael reaction catalyzed by DERA‐WT and DERA‐MA, respectively.

The archetypical class I aldolase DERA has been extensively applied in biocatalysis owing to its ability to construct C−C bonds. Notable industrial examples include the rapid synthesis of the statin side chain, and more recently the integration of engineered DERA in the biocatalytic cascade synthesis of Islatravir.[^59^, ^60^, ^61^] DERA naturally catalyzes the asymmetric aldol reaction of acetaldehyde (**9**) to D‐glyceraldehyde‐3‐phosphate (**10**) to yield 2‐deoxy‐D‐ribose‐5‐phosphate (**11**, Figure 3). Mechanistically, DERA uses a catalytic lysine (Lys‐167) to form a Schiff base with the aldol donor, generating a reactive enamine species upon deprotonation. Previous works have largely focused on substrate scope expansion of the DERA‐catalyzed aldol reaction to include other aldehydes or ketones.[^62^, ^63^] Kunzendorf et al. have recently demonstrated that the catalytic machinery of DERA from *E. coli* can also give rise to iminium catalysis, promoting the Michael addition of **3** to **4** (Figure 3).^[64]^ The low promiscuous Michaelase activity identified in DERA wild‐type was optimized by directed evolution combining focused and random mutagenesis (11 rounds) leading to a 190‐fold increase of the catalytic activity. The final variant DERA‐MA has 12 amino acid substitutions and catalyzes the Michael addition of **3** to **4** with a *k*_cat_ of 0.38 s^−1^. The high‐resolution crystal structure of DERA‐MA revealed the formation of an iminium ion between the carbonyl substrate **4** and the catalytic Lys‐167. Directed evolution also helped to redesign two loops to create a binding pocket for the covalently bound substrate. For preparative‐scale synthesis, DERA‐MA can be used at a similar catalyst loading as 4‐OT(F11), achieving excellent conversion and good isolated product yield (Table 1). This work paves the way for engineering this class I aldolase to catalyze other mechanistically related and synthetically useful reactions. Moreover, other class I aldolases, such as DHAP/DHA‐dependent enzymes,^[65]^ could also be promising templates for iminium catalysis.

## 4 Computationally Designed and Evolved Enzymes

**Figure 4:**
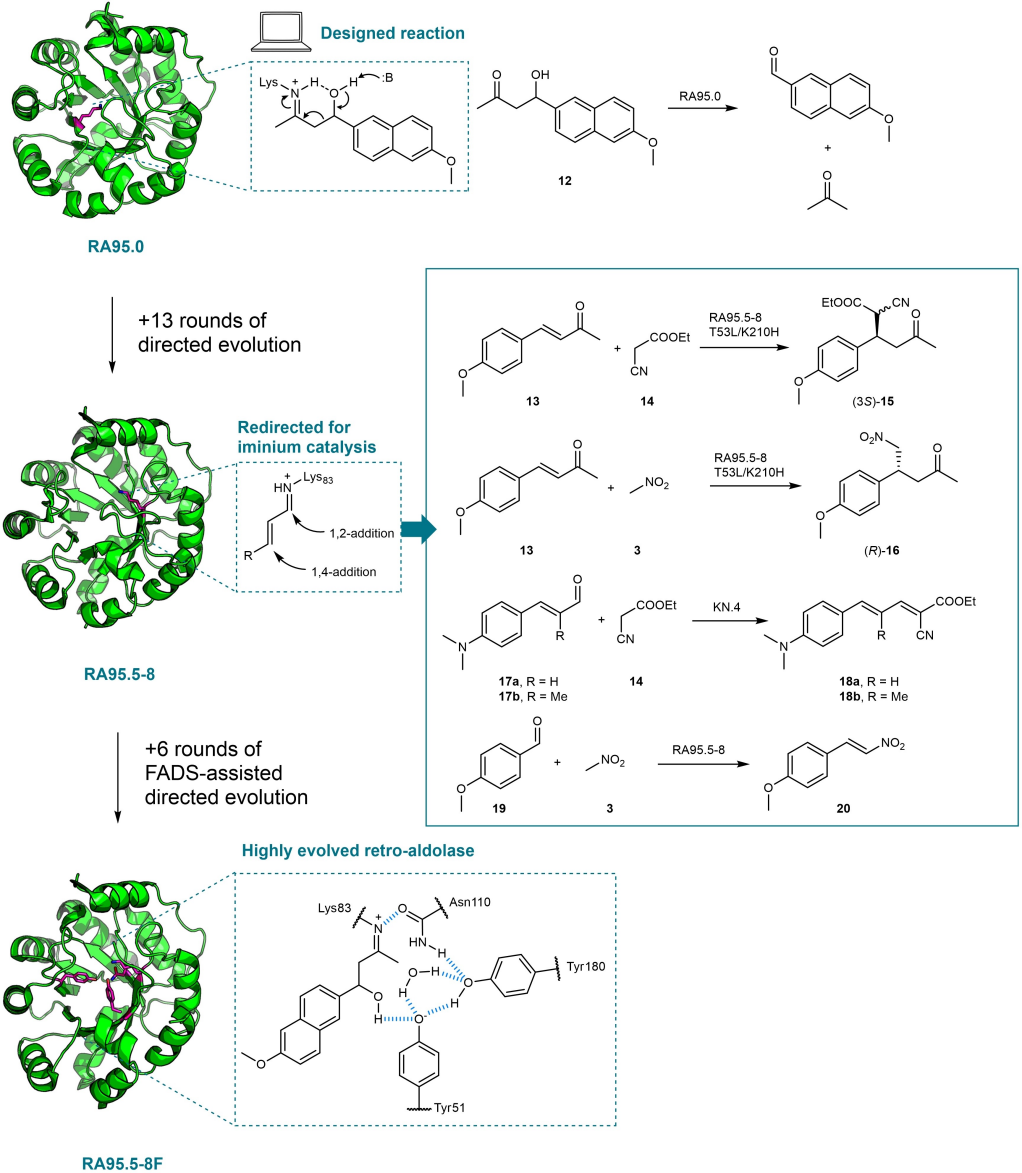
The design and evolution of RA95.0 enzyme variants. RA95.5‐8 was redirected for various iminium catalysis reactions. FADS: fluorescence‐activated droplet sorting.

Computational enzyme design is a promising tool for the creation of new biocatalysts. Notable designs include Kemp eliminase,^[66]^ retro‐aldolase,^[67]^ and Diels–Alderase.^[68]^ Although the desired activities have been obtained, the catalytic efficiency of computationally designed enzymes often falls many orders of magnitude behind that of natural enzymes. With the help of directed evolution, these low‐level activities can be substantially enhanced.[^69^, ^70^] The computationally designed retro‐aldolase RA95.0 is a case in point. RA95.0 was originally designed by introducing 11 amino acid substitutions into a TIM barrel scaffold protein (PDB code: 1LBL) to catalyze the retro‐aldol cleavage of methodol **12** using a catalytic lysine for Schiff base formation, in combination with an ordered water molecule and a hydrophobic substrate‐binding pocket (Figure 4).^[71]^ The initially designed enzyme was subjected to 13 rounds of directed evolution, which led to the identification of variant RA95.5‐8 with over 4400‐fold improved catalytic efficiency and, surprisingly, a completely remodeled active site in which the key catalytic lysine was switched to a different position (from K210 to K83).^[72]^ Subsequently, RA95.5‐8 was optimized further by another 6 rounds of directed evolution empowered by a ultrahigh‐throughput microfluidic fluorescence‐activated droplet sorting (FADS) screening method to obtain a highly active retro‐aldolase RA95.5‐8F with an activity that rivals that of natural enzymes (Figure 4).^[73]^

Hilvert and co‐workers have found that the catalytic apparatus of RA95.5‐8 displays remarkable catalytic promiscuity towards mechanistically related carboligations (Figure 4). The enzyme was investigated for the Michael addition of various nucleophiles to α,β‐unsaturated ketone **13**.^[74]^ Initially, RA95.5‐8 was found to efficiently promote the Michael addition of **14** to **13** via an iminium ion intermediate formed between the catalytic Lys83 and **13**, yielding **15** with good enantiocontrol at the site of addition. Site‐saturation mutagenesis led to variant RA95.5‐8(T53L/K210H) with approximately threefold improvement in catalytic efficiency.^[74]^ Next, nitroalkanes such as nitromethane were also found to be good nucleophiles for the Michael reactions using RA95.5‐8, giving access to important chiral synthons (γ‐nitroketones).^[75]^ Screening of a panel of retro‐aldolases suggested the previously identified variant RA95.5‐8(T53L/K210H) was the best candidate for the nitromethane addition, achieving up to 2000 TTN for the Michael addition of **3** to **13** (Table 1). Interestingly, RA95.5‐8 was also found to promote the Michael‐type addition of acetone to nitrostyrene, generating the opposite enantiomer of the desired γ‐nitroketone.^[75]^ It is worth noting that the highly evolved retro‐aldolase variant RA95.5‐8F^[73]^ (Figure 4) does not possess a higher catalytic rate for the Michael reaction compared to its ancestor RA95.5‐8, indicating RA95.5‐8F was highly specialized for the aldol reaction due to the intensive FADS‐assisted directed evolution. More recently, Garrabou et al. also demonstrated that RA95.5‐8 is malleable for stereodivergent evolution, resulting in four variants promoting stereocomplementary Michael reactions with good stereo‐control.^[76]^

In addition to conjugate additions, the catalytic site of RA95.5‐8 can be channeled to furnish 1,2‐additions (Figure 4). When using α,β‐unsaturated aldehydes (**17**) instead of ketones, biocatalytic Knoevenagel condensation was achieved.^[77]^ The reaction also proceeds with the formation of an enzyme‐bound iminium ion, but the subsequent addition of the nucleophile **14** occurs at the carbonyl carbon. The Knoevenagel condensation product is then generated by the β‐elimination of the intermediate after nucleophilic addition. To further augment the Knoevenagel condensation activity, RA95.5‐8 was subject to directed evolution, assisted by an agar plate colorimetric screening method based on the formation of the red‐colored product **18**. After four rounds of directed evolution, the best variant named KN.4 emerged with a *k*_cat_ of 7.1 s^−1^ for the conversion of **17** **b** to give **18** **b**.^[77]^ Similarly, the 1,2‐addition reactivity of RA95.5‐8 was also exploited for the synthesis of nitroolefins by the Henry condensation of nitromethane to 4‐MeO‐benzaldehyde (**19**, Figure 4).^[78]^ Notably, enzymatic Henry coupling reactions (no dehydration) were reported earlier which proceed through hydrogen bond activation of the aldehyde to generate β‐hydroxynitro compounds.^[79]^ By contrast, RA95.5‐8 catalyzes Henry condensation via the nucleophilic 1,2‐addition of the lysine‐bound iminium ion followed by β‐elimination of the intermediate, leading to a nitroolefin.

## 5 Artificial Enzymes

Remarkably, nature can already achieve vastly diverse chemistry to support life with a small set of 20 canonical amino acids. However, reactions that cannot be catalyzed by the functionalities present in the canonical amino acids are also regularly found in nature. This is made possible by either post‐translational modification of specific amino acids or the recruitment of cofactors, for example, the autocatalytic formation of electrophilic MIO (4‐methylideneimidazole‐5‐one)^[80]^ in phenylalanine ammonia lyases and the employment of flavins and heme cofactors to support various chemistries. Similarly, both strategies have been utilized for the development of artificial enzymes incorporating either non‐canonical amino acids or non‐natural cofactors. Recently, iminium biocatalysts based on artificial enzymes containing a catalytic amine have also been developed. Different from the natural or computationally designed enzymes discussed above, which use a canonical amino acid (proline or lysine) as the key catalytic amine, the development of artificial enzymes gives the freedom to explore other non‐natural catalytic amine functionalities. Two examples are discussed below: LmrR which uses a genetically expanded non‐canonical catalytic amine and a biotin–streptavidin‐based hybrid catalyst that harbors a non‐natural synthetic amine as the cofactor.

### 5.1 LmrR

LmrR belongs to the multidrug resistance regulators (MDRs) which are involved in the recognition and binding of foreign compounds such as antibiotics. It is a homodimeric protein with a large hydrophobic pore at the dimer interface.^[81]^ LmrR's promiscuous binding property prompted Roelfes and co‐workers to select this protein as a scaffold for the construction of various artificial enzymes.^[82]^ Several techniques have been applied to install functional groups for catalysis into the LmrR scaffold. For instance, earlier efforts utilized the bioconjugation approach to covalently incorporate a Lewis acidic Cu^II^ complex to facilitate various Lewis acid catalytic reactions.[^83^, ^84^] Alternatively, a similar Cu^II^ complex can also be recruited to the hydrophobic core of LmrR via supramolecular assembly to a “hydrophobic clamp” formed between two symmetrically aligned tryptophan residues.[^85^, ^86^] Recently, the emergence of technologies for the genetic incorporation of non‐canonical amino acids (ncAAs) has opened the gateway for the creation of full gene‐encoded artificial enzymes.[^87^, ^88^] Using this technology, novel artificial enzymes based on the LmrR scaffold containing a ncAA have also been created to enable iminium biocatalysis.

**Figure 5:**
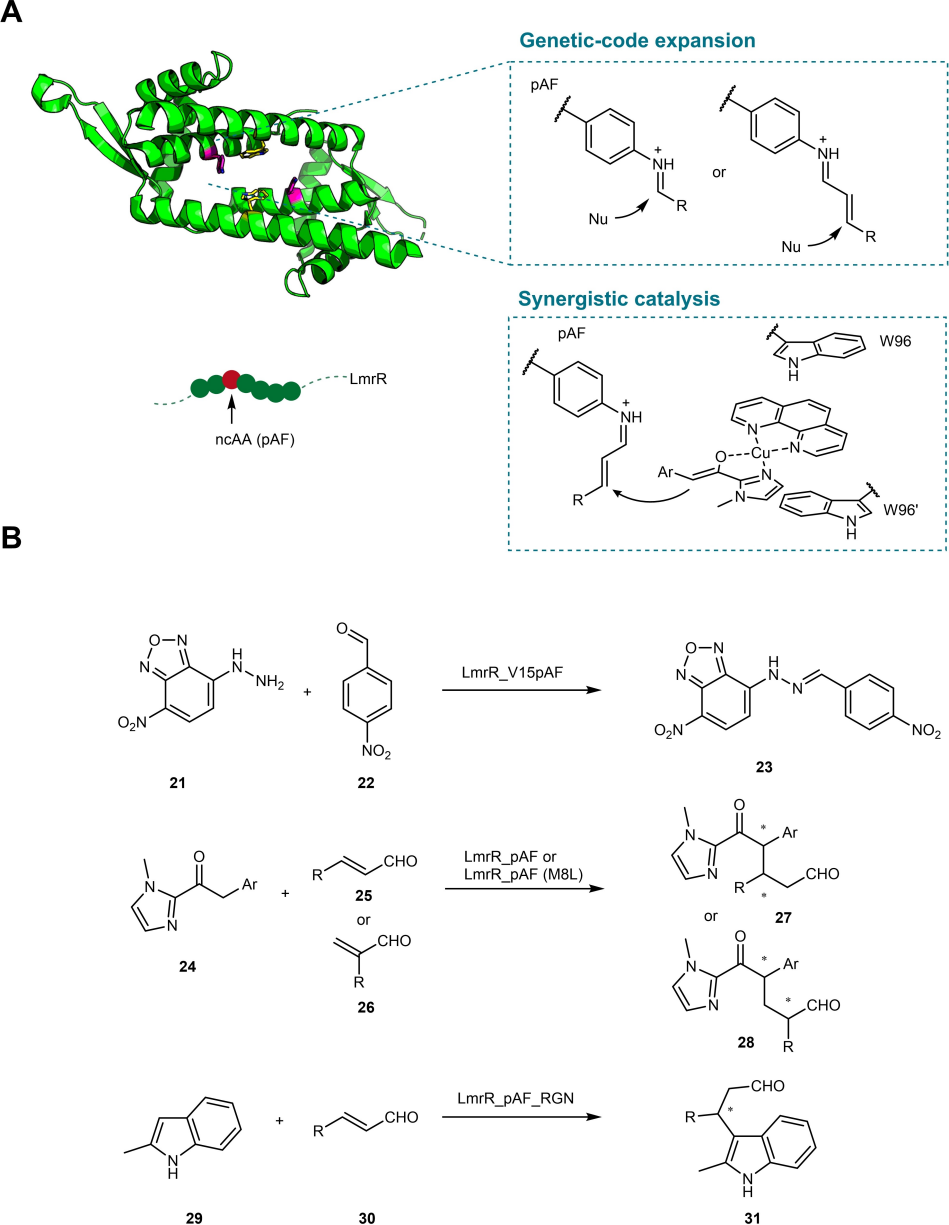
A) Artificial enzyme LmrR_V15pAF with a catalytic aniline side‐chain, constructed using genetic‐code expansion technology. B) LmrR_V15pAF catalyzed reactions proceed via iminium ion intermediates.

Drienovska et al. reported the biocatalytic hydrazone and oxime formation catalyzed by an artificial enzyme featuring an unnatural catalytic aniline residue (Figure 5).^[89]^ The ncAA selected was *p‐*aminophenylalanine (pAF), which unlike the N‐terminal proline or lysine discussed above, is an aromatic amine that may also form an enzyme‐bound iminium ion. However, it was realized that for LmrR, the direct incorporation of *p‐*aminophenylalanine (pAF) using the amber stop codon suppression method^[90]^ was problematic. An indirect approach was used instead, in which ncAA *p‐*azidophenylalanine (pAzF) was first genetically incorporated into LmrR, followed by a Staudinger reduction of the azide to the desired amine group. Using this approach, LmrR variant LmrR_V15pAF (V15 mutated to ncAA pAF) was found to be the best candidate for the hydrazone formation between **21** and **22** (Figure 5B), displaying catalytic activity more than two orders of magnitude higher compared to simple aniline (and its derivatives) in the reaction buffer. Yet, the catalytic power contributed by pAF in LmrR_V15pAF is quite modest because replacing the pAF with tyrosine resulted in only a 10‐fold activity reduction.^[91]^ Hence, directed evolution was performed to optimize the role of pAF for catalyzing the hydrazone formation reaction. Iterative site‐saturation mutagenesis identified LmrR_pAF RMHL with almost 100‐fold improvement in *k*_cat_ (from 5×10^−4^ to 2.76×10^−2^ s^−1^). The mutations greatly strengthen the role of pAF for catalysis, with the knockout mutant (pAF mutate to tyrosine) possessing negligible activity (200‐fold reduction).^[91]^

To expand the reaction scope, Zhou and Roelfes combined the iminium activation enabled by pAF with metal catalysis to achieve synergistic enantioselective Michael addition of **24** to **25** (Figure 4).^[92]^ The metal catalyst Cu^II^ phenanthroline was recruited to the catalytic site via hydrophobic interactions (“sandwiched” between two active‐site tryptophan residues) to achieve the activation of the nucleophile **24**. The activated nucleophile is then positioned to undergo conjugation addition to the enzyme‐bound iminium ion formed between the aniline side chain and **25** (Figure 5A). Removing either the ncAA pAF or the metal catalyst completely abolished the activity, indicating both components are needed for catalysis. Screening of several mutants resulted in the identification of LmrR_pAF M8L which showed higher activity towards this reaction. The substrate scope of both LmrR_pAF and LmrR_pAF M8L was explored (eight examples), demonstrating good enantioselectivity and diastereoselectivity for these artificial enzymes.^[92]^ In a follow‐up study, α‐substituted acrolein **26** was also tested as the Michael acceptor in this synergistic catalysis mode, affording **28** with good enantioselectivity on both chiral centers, indicating the catalytic system has excellent enantiocontrol over the protonation step of the Michael addition reaction (Figure 5B).^[93]^ Furthermore, LmrR_pAF was also used as a template to develop a Friedel–Crafts alkylase.^[94]^ In this case, the synergistic system is not required since the indole nucleophile **29** itself is sufficiently activated. Protein engineering identified a triple‐substituted variant LmrR_pAF_RGN with improved Friedel–Crafts alkylase activity, giving access to **31** with moderate to good enantioselectivity (Figure 5B).

### 5.2 Streptavidin

**Figure 6:**
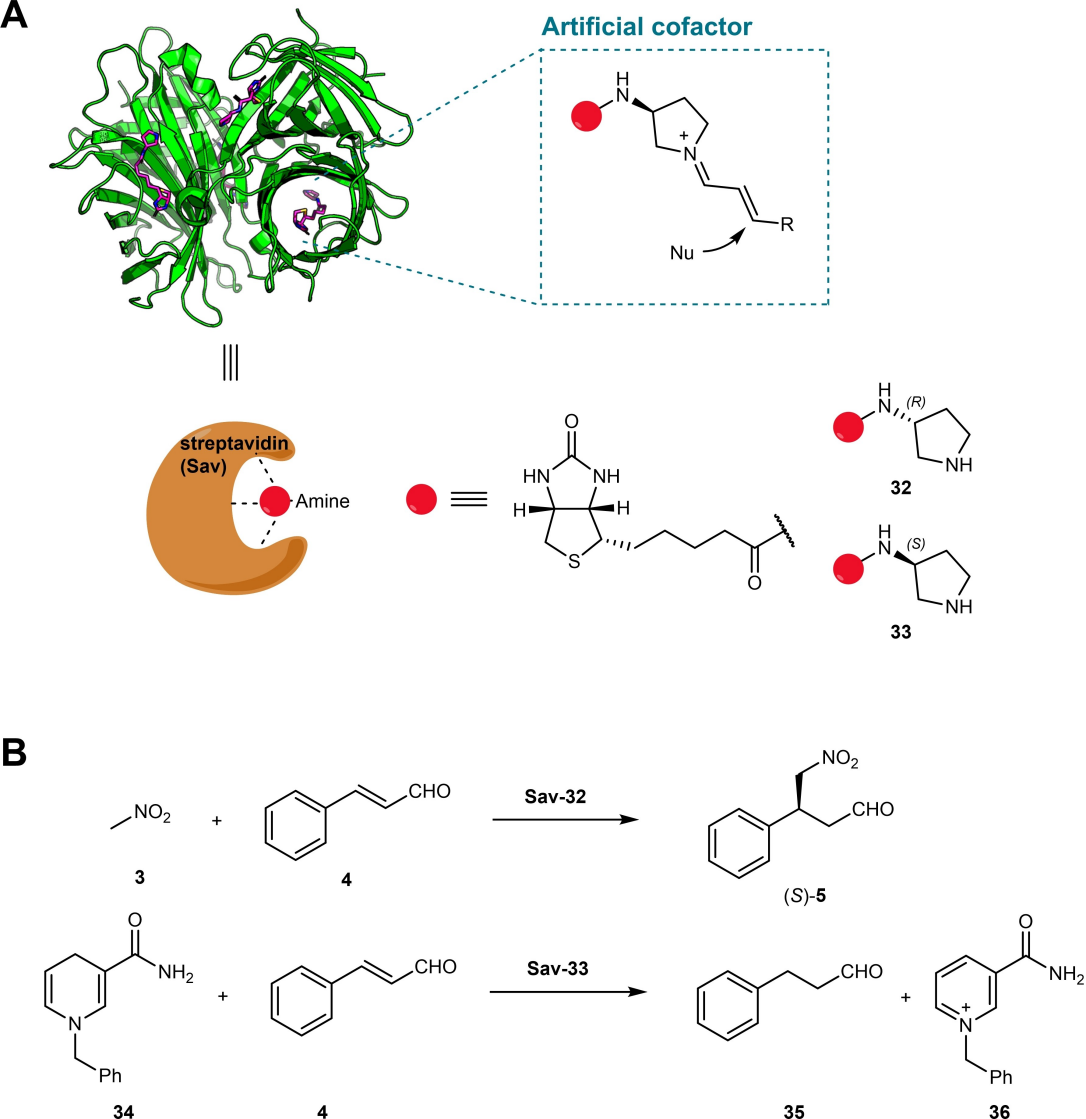
A) Hybrid catalyst based on the streptavidin–biotin technology; the biotin was functionalized with an amine to accommodate iminium catalysis. B) Michael addition and ene reduction catalyzed by artificial hybrid catalysts.

Streptavidin (**Sav**) and biotin form one of the strongest non‐covalent interactions in nature (Figure 6). This interaction has been exploited in many biological applications such as western blotting and immunoassays.^[95]^ Streptavidin has also been used as a platform for the creation of artificial enzymes. Pioneered by the Ward group, artificial metalloenzymes (ArMs) that use streptavidin as the host protein and functionalized biotin as guest molecules were constructed to catalyze non‐natural reactions such as olefin metathesis and transfer hydrogenations.[^96^, ^97^] The biotin was functionalized with a chemical moiety that anchors the catalytic metal, whereas the protein host can assist in tuning both the activity and selectivity. Thus, the modified biotin molecule can also be treated as an artificial “cofactor”.

More recently, biotin–streptavidin technology has been further developed to promote iminium catalysis. Nödling et al. have reported the asymmetric Michael addition of nitromethane to cinnamaldehyde catalyzed by a biotin–streptavidin‐based hybrid catalyst (Figure 6).^[98]^ A set of secondary amine organocatalysts covalently attached to biotin were chemically synthesized and screened as guest molecules to accommodate iminium catalysis in streptavidin. Biotinylated aminocatalyst **32** was found to be the best guest molecule for catalysis (Figure 6). Without the host protein, **32** catalyzed the Michael reaction of **3** to **4** poorly in buffer and afforded racemic product. The addition of **Sav** enhanced the catalytic rate by fivefold and induced good product enantioselectivity. After the reaction condition optimization, 1 mol % hybrid catalyst **Sav**‐**32** can promote the Michael addition of **3** to **4**, yielding (*S*)‐**5** with good conversion (80 %) and enantioselectivity (82 % *ee*, Table 1). Crystallography and molecular dynamic simulation studies supported the iminium ion catalytic mechanism and the product stereochemistry outcome. This hybrid catalyst also accepts a range of cinnamaldehyde derivatives, yielding the corresponding Michael addition products with acceptable to good conversion and enantioselectivity.^[98]^ Additionally, the same hybrid catalyst system has been applied to create an artificial ene‐reductase (ERED) to achieve the C=C bond reduction of cinnamaldehyde (Figure 6).^[99]^ Natural EREDs or DBRs (double‐bond reductases) usually activate their substrate via hydrogen bonding and the hydride donors are cofactors such as NAD(P)H or flavins. Given that streptavidin does not have a dedicated binding site for these natural cofactors, the alternative cofactor mimic dihydrobenzyl nicotinamide (BNAH, **34**) was sought as the hydride source. Analytic‐scale reactions showed **Sav‐33** can perform effective reductions of various cinnamaldehyde derivatives.^[99]^ However, this hybrid catalyst has not yet been subjected to directed evolution to improve its catalytic efficiency and selectivity.

## 6 Summary and Outlook

Iminium catalysis, a fundamental branch of organocatalysis, has been integrated into biocatalysis by enzyme designers and engineers, creating various novel biocatalysts that promote reactions that are new to nature but beneficial to humans. Natural enzymes or computationally designed enzymes containing either a catalytic lysine or N‐terminal proline have been redirected for iminium biocatalysis. Alternatively, many successful examples have also been found with artificial enzymes bearing a synthetic amine cofactor or a non‐canonical amino acid enabled by genetic‐code expansion. Inspired by the many reactions enabled by iminium catalysis, novel enzymes have been created to facilitate various reactions including Michael addition, epoxidation, cyclopropanation, Knoevenagel condensation, Henry condensation, transfer hydrogenation, Friedel–Crafts alkylation, and hydrazone/oxime formation. With directed evolution as a unique biocatalyst optimization method, superior enzymes can be evolved that greatly outperform their small‐molecule catalyst counterparts. Moreover, these iminium biocatalysts are genetically encoded (except in the hybrid catalyst case), allowing facile and environmentally friendly catalyst production via fermentation, bypassing the need for organic synthesis of the catalysts.

**Figure 7:**
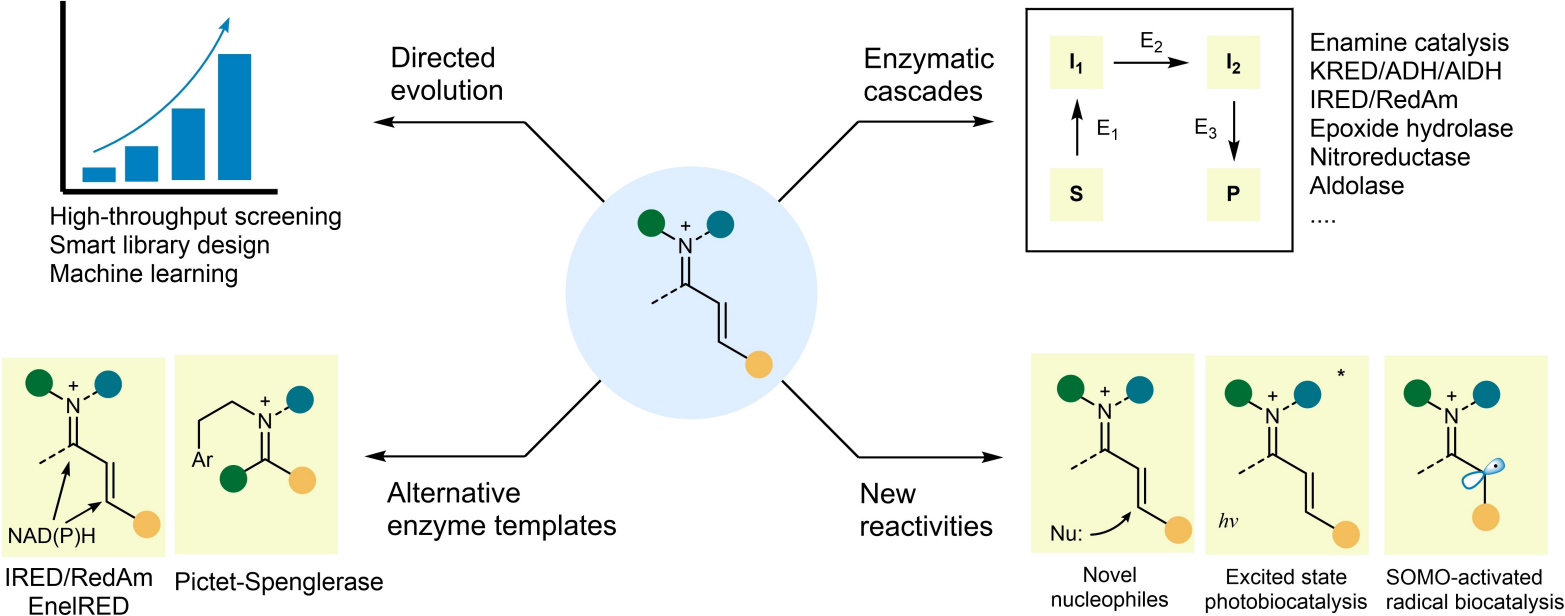
Future perspectives of iminium biocatalysis. KRED (ketoreductase), ADH (alcohol dehydrogenase), AlDH (aldehyde dehydrogenase), IRED (imine reductase), RedAm (reductive aminase), EnelRED (multifunctional imine reductase),^[100]^ SOMO (singly occupied molecular orbital).

Yet several issues require more attention for iminium biocatalysis to reach broader applicability. A major drawback of iminium catalysis with small molecules is the high catalyst loading (typically 10–20 mol %). Although many iminium biocatalysts discussed herein can be used at lower catalyst loadings (typical 0.5–5 mol %, minimum 0.05 mol %, Table 1), their activities still do not reflect the true power of enzymes and considering the large molecular weight of enzymes, the applied high weight percentage of the biocatalyst is not an advantage. However, this limitation can be resolved by the ever more efficient protein engineering methods to boost the initial activities, as demonstrated by the directed evolution of fused 4‐OT, DERA, and RA95.5‐8 which resulted in highly efficient iminium biocatalysts. To further improve the catalytic efficiency using directed evolution, (ultra)high‐throughput screening methods such as FADS may be needed to obtain an enzyme with a sophisticated hydrogen‐bond network tailored to iminium catalysis (Figure 7). In addition, the substrate/product concentrations of the reactions reported here are also usually low (typically in the millimolar range, Table 1), which is unsuitable for practical, large‐scale synthesis. We anticipate that further enzyme engineering combined with reaction engineering can help to solve these issues.

Nevertheless, we are confident that the recent development of iminium biocatalysis has just scratched the surface. In addition to using directed evolution for improving existing enzymes, alternative enzymes such as IRED/RedAm/EnelRED[^100^, ^101^] and Pictet–Spenglerases^[25]^ where iminium ions are used could be hijacked for substrate‐assisted iminium catalysis or multicomponent reactions (Figure 7). New reaction patterns based on iminium catalysis will surely be engineered into enzymes. Since the reactions proceed in aqueous solvent, water‐compatible nucleophiles which are not suitable to use in traditional organocatalysis in organic solvent but are unique to the biocatalytic realm may be used for discovering completely new reactivity (Figure 7). The complex protein environment may also result in novel reactivities not seen in small‐molecule catalysts. Iminium biocatalysis can also be incorporated into enzymatic cascades to efficiently synthesize target compounds in an economic one‐pot fashion. Furthermore, as already demonstrated by the synergistic catalysis of LmrR, the combination of iminium catalysis with even more distinct modes of catalysis, for example, photocatalysis[^102^, ^103^] and SOMO‐activated radical chemistry,^[104]^ can spark the development of new enzymes (Figure 7). Finally, with the help of more powerful tools for designing and engineering proteins, and with the increasing knowledge of protein structure and enzyme catalysis, industrially applicable highly active enzymes for iminium catalysis may be realized in the near future.

## Conflict of interest

The authors declare no conflict of interest.

## Biographical Information

*Guangcai Xu obtained his Master's degree from the University of Liverpool (UK) in 2013. Afterward, he moved to Suzhou (China) to work on industrial biocatalysis. From 2017–2021, he pursued his doctoral degree in the group of Prof. Gerrit J. Poelarends at the University of Groningen (The Netherlands). During this period, he developed enzymes for iminium biocatalysis. Currently, he is working with Prof. Jason Micklefield at the University of Manchester (UK) as a postdoctoral research associate*.

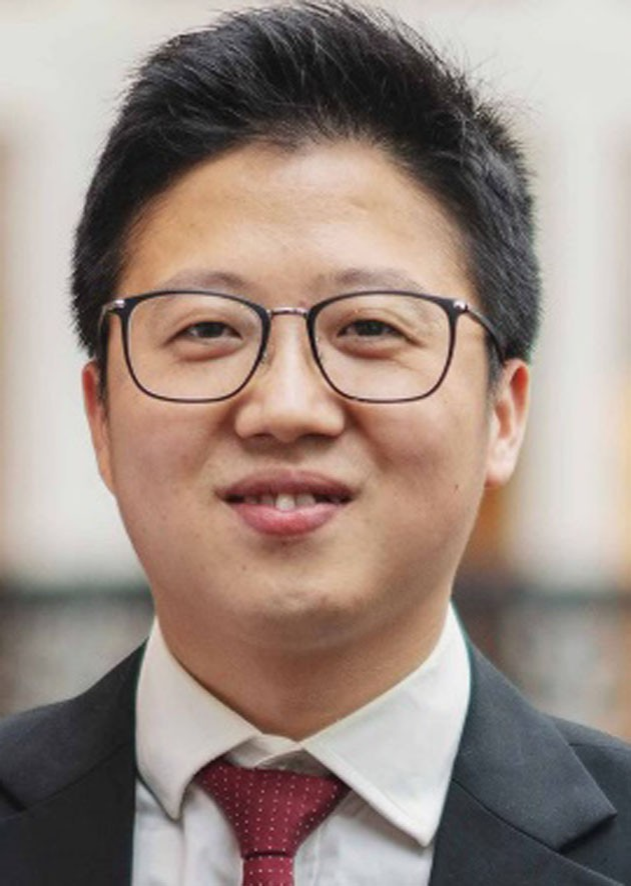

## Biographical Information

*Gerrit J. Poelarends has been leading an independent research group at the department of Chemical and Pharmaceutical Biology, University of Groningen (The Netherlands) since 2006, with promotion to Full Professor of Pharmaceutical Biotechnology in 2017. His research interests are in the enzyme engineering and biocatalysis fields with a focus on the discovery and exploitation of promiscuous enzyme activities for the creation of novel biocatalysts capable of promoting new‐to‐nature transformations*.

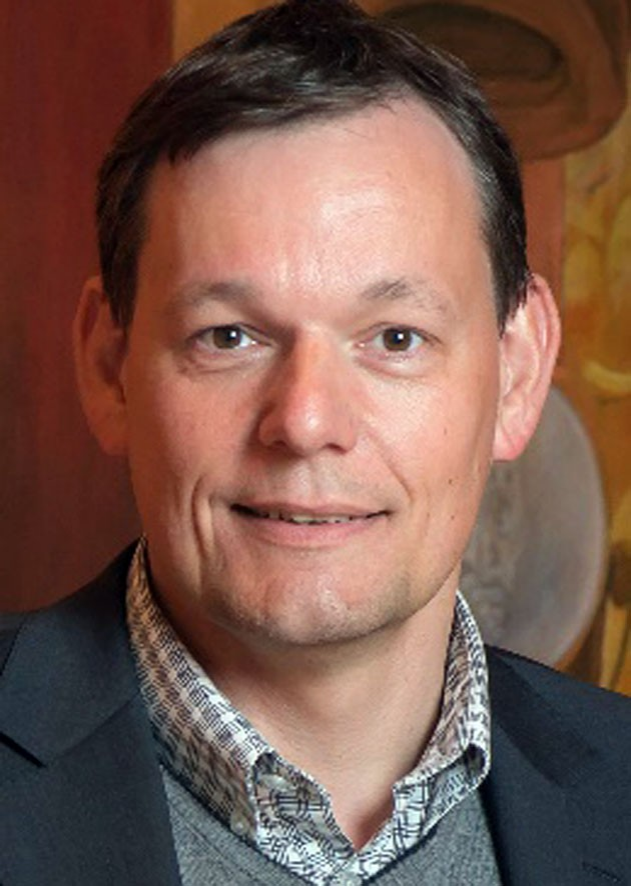
